# Structural Basis for Disparate Sugar-Binding Specificities in the Homologous Cargo Receptors ERGIC-53 and VIP36

**DOI:** 10.1371/journal.pone.0087963

**Published:** 2014-02-03

**Authors:** Tadashi Satoh, Kousuke Suzuki, Takumi Yamaguchi, Koichi Kato

**Affiliations:** 1 Graduate School of Pharmaceutical Sciences, Nagoya City University, Mizuho-ku, Nagoya, Japan; 2 JST, PRESTO, Mizuho-ku, Nagoya, Japan; 3 Okazaki Institute for Integrative Bioscience and Institute for Molecular Science, National Institutes of Natural Sciences, Myodaiji, Okazaki, Aichi, Japan; NCI-Frederick, United States of America

## Abstract

ERGIC-53 and VIP36 are categorized as leguminous type (L-type) lectins, and they function as cargo receptors for trafficking certain *N*-linked glycoproteins in the secretory pathway in animal cells. They share structural similarities in their carbohydrate recognition domains (CRDs) but exhibit distinct sugar-binding specificities and affinities. VIP36 specifically interacts with the α1,2-linked D1 mannosyl arm without terminal glucosylation, while ERGIC-53 shows a broader specificity and lower binding affinity to the high-mannose-type oligosaccharides, irrespective of the presence or absence of the non-reducing terminal glucose residue at the D1 arm. In this study, we determined the crystal structure of ERGIC-53–CRD in complex with their binding partner, MCFD2 and the α1,2 mannotriose which corresponds to the trisaccharide of the D1 arm of high-mannose-type glycans. ERGIC-53 can interact with the D1 trimannosyl arm in two alternative modes, one of which is similar but distinct from that previously observed for VIP36. ERGIC-53 has a shallower sugar-binding pocket than VIP36 because of the single amino acid substitution, Asp-to-Gly. This enables ERGIC-53 to accommodate the non-reducing terminal glucose of the D1 arm in its CRD. In the other interaction mode, the 3-OH group of the terminal mannose was situated outward with respect to the sugar binding pocket, also enabling the Glcα1-3 linkage formation without steric hindrance. Our findings thus provide a structural basis for the broad sugar-binding specificity of the ERGIC-53/MCFD2 cargo receptor complex.

## Introduction

*N*-linked oligosaccharides play important roles in the determination of glycoprotein fates in cells through interactions with a series of intracellular lectins [1], [2], [3], [4], [5], [6], [7]. These lectins specifically recognize partially trimmed processing intermediates of high-mannose-type oligosaccharides presented on the target polypeptide chain and thereby regulate protein folding, degradation, and transport. The sugar chain is initially introduced by oligosaccharyltransferase as a triglucosyl high-mannose-type tetradecasaccharide (Glc_3_Man_9_GlcNAc_2_) in the endoplasmic reticulum (ER). The *N*-linked glycans displayed on the nascent polypeptide chain are trimmed by a series of glucosidases and mannosidases. Glucosidase I cleaves the outermost α1,2-linked glucose residue at the D1 arm. Subsequently, glucosidase II removes the second and third α1,3-linked glucose residues. During the folding process in the ER, incompletely folded glycoproteins are subjected to re-glucosylation by the action of the folding sensor enzyme, UDP-glucose:glycoprotein glucosyltransferase (UGGT). The ER chaperone-like lectins calnexin and calreticulin specifically interact with the monoglucosylated glycoforms and thereby assist the folding of the carrier proteins.

After correct folding and assembly in the ER, the *N*-linked glycoproteins are transported to the Golgi apparatus *via* the ER-Golgi intermediate compartment (ERGIC) by vesicular transport. Incorporation of the cargo glycoproteins into the transport vesicles is mediated by transmembrane cargo receptors, including ERGIC-53 and VIP36 [8], [9]. These cargo receptors are termed leguminous type (L-type) lectins because they share homologous carbohydrate recognition domains (CRDs) with structural resemblance to leguminous lectins such as concanavalin A [10]. ERGIC-53 (ER–Golgi intermediate compartment protein of 53 kDa), the most popular marker for the ERGIC, functions as a cargo receptor between the ER and ERGIC for certain glycoproteins, including the lysosomal glycoproteins cathepsin Z and cathepsin C and blood coagulation factors V and VIII [11], [12]. ERGIC-53 consists of a CRD, a stalk domain, and a transmembrane region along with a short cytoplasmic segment. The luminal stalk domain following the CRD forms coiled-coil helices and membrane-proximal intermolecular disulfide bridges thereby mediating dimerization or hexamerization [11], [13]. However, biological role of this stalk domain remains largely unknown, since binding of ERGIC-53 to its cargo glycoprotein cathepsin Z or immobilized mannose was not affected by its oligomerization states of ERGIC-53 [11], [13]. It has been shown that ERGIC-53 forms a complex with MCFD2 (multi-coagulation factor deficiency 2), a 16 kDa co-receptor possessing two EF-hand Ca^2+^-binding motifs [14], [15] and thereby operates as a cargo receptor specific for factor V and factor VIII [12], [16]. Unlike ERGIC-53, VIP36 is distributed to either the pre-Golgi early secretory pathway [17], [18] or post-Golgi pathway [8], [19], whereas VIPL (VIP36-like lectin) acts as a non-cycling ER-resident protein [20]. We previously elucidated the sugar-binding properties of these three L-type lectins, ERGIC-53, VIP36, and VIPL using frontal affinity chromatography (FAC) [21], [22]. The FAC data demonstrated that VIP36 and VIPL interact with high-mannose-type oligosaccharides having deglucosylated α1,2-linked trimannose in the D1 arm, whereas ERGIC-53 exhibits a lower binding affinity and broader specificity not discriminating between monoglucosylated and deglucosylated glycoforms. Therefore, these L-type lectins have distinct cellular localizations and sugar-binding specificities, suggesting their disparate functions in the secretory pathway despite the structural similarities of their CRDs [22]. It has been shown that ERGIC-53 along with several chaperone proteins is upregulated in ER stress conditions such as tunicamycin treatment, suggesting that the upregulation of this lectin promotes the export capacity of glycoprotein cargos in the case of an emergency [23], [24], [25].

To gain structural insights into the functional roles of these L-type lectins in the sorting and trafficking of secretory glycoproteins, crystal structures for the CRDs of rat ERGIC-53 with [26] and without Ca^2+^ [27], human ERGIC-53 complexed with MCFD2 [28], [29], and canine VIP36 with and without Ca^2+^ and mannosyl ligands [30] have been solved, confirming their structural similarity to the leguminous lectins. In particular, the crystal structures of the VIP36 CRD complexed with mannosyl di- and tetrasaccharide ligands revealed that the D1 branch of high-mannose-type oligosaccharides is accommodated on the concave β-sheet involving Ca^2+^-binding loops [30]. It has also been shown that MCFD2 but not ERGIC-53–CRD undergoes significant conformational changes upon their interaction [28], [29]. The data provided a working model of cooperative interplay between ERGIC-53 and MCFD2 in interactions with FV and FVIII; MCFD2 binds the polypeptide segments of these coagulation factors, whereas ERGIC-53 captures their *N*-linked glycans. Although crystallographic data has recently been reported for ERGIC-53–CRD in complex with α1,2-linked mannobiose (termed α2-Man_2_) [31], the way in which this lectin shows a broad specificity toward monoglucosylated high-mannose-type oligosaccharides remains elusive because position of the non-reducing terminal Man(D1) residue could not be unambiguously determined in the crystal structure.

**Figure 1:**
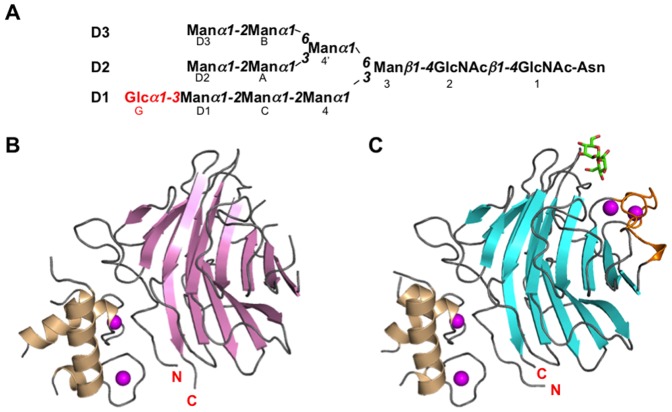
Overall structures of binary and ternary complexes of ERGIC-53–CRD. (A) Schematic representation of Glc_1_Man_9_GlcNAc_2_ showing the nomenclature of oligosaccharide residues and branches. Glucose residue is shown in red. Ribbon models of binary and ternary complexes of ERGIC-53–CRD are shown in (B) and (C), respectively. Ca^2+^-free ERGIC-53–CRD and Ca^2+^/α2-Man_3_-bound ERGIC-53–CRD are colored pink and cyan, respectively. MCFD2 is shown in wheat. The bound α2-Man_3_ and Ca^2+^ ions are shown as green stick and magenta sphere models, respectively. In the trimannosyl ligand, the two mannose residues giving unambiguous electron densities were drawn. Ca^2+^-binding loops (residues 155–161 and 176–185) are highlighted in orange in (C).

In this study, we present new crystal structures of ternary complexes of ERGIC-53–CRD formed with MCFD2 and α2-Man_2_ or α1,2-linked mannotriose (termed α2-Man_3_) corresponding to Man(D1)-Man(C)-Man(4); Figure 1A. The data provide structural insight into the broad sugar-binding specificity of the cargo receptor ERGIC-53 in comparison with VIP36.

## Results and Discussion

### Overall structure of the ERGIC-53–CRD/MCFD2/α2-Man_3_ ternary complex

To observe the electron density corresponding to the non-reducing end Man(D1) residue, which could not be interpreted in the recently reported crystal structure of ERGIC-53–CRD complexed with α2-Man_2_ [31], we attempted to discover other crystallization conditions in the presence of MCFD2. We previously determined the 1.84-Å crystal structure of the binary complex of human ERGIC-53–CRD (residues 31–269) and MCFD2 (residues 27–146) [28]. In this study, we tried soaking experiments using the crystals with α2-Man_2_ and α2-Man_3_. However, the electron density of any sugar ligands could not be identified in this *P*3_1_21 crystal structure. Hence, we performed further crystallization screening using a different construct of MCFD2 (residues 67–146), and eventually obtained well-diffracting crystals with a different crystal form (space group *C*2). The *C*2 crystal structure was solved by the molecular replacement method with the previously reported binary ERGIC-53–CRD/MCFD2 complex (Protein Data Bank code 3A4U) [28] as a search model. The final model of the binary complex was refined to a resolution of 1.80 Å has an *R*_work_ of 17.4% and *R*_free_ of 20.2% (Table 1). The Ca^2+^-binding loops (residues 155–161 and 176–185) of ERGIC-53-CRD and the loop comprised of residues 98–110 of MCFD2 along with their N- and C-terminal segments gave no interpretable electron density. Although ERGIC-53–CRD and MCFD2 both possess two Ca^2+^-binding sites, Ca^2+^ ions were missing in the ERGIC-53–CRD crystal structure. This occurred although 1 mM CaCl_2_ was contained in the crystallization buffer, while MCFD2 held the two Ca^2+^ ions (Figure 1B). This suggests that ERGIC-53–CRD is apt to lose Ca^2+^ ions in comparison with MCFD2.

**Table 1:** Data collection and refinement statistics for binary and ternary complexes of ERGIC-53–CRD.

	ERGIC-53-CRD/MCFD2	ERGIC-53-CRD/MCFD2/α2-Man_2_	ERGIC-53-CRD/MCFD2/α2-Man_3_
Crystallographic data			
Space group	*C*2	*C*2	*C*2
Unit cell *a*/*b*/*c* (Å)	104.0/59.8/55.1	101.7/58.7/56.4	100.3/58.2/55.4
β (°)	104.9	109.7	109.3
Data processing statistics			
Beam line	PF-AR NE3A	PF-AR NW12A	PF BL5A
Wavelength (Å)	1.00000	1.00000	1.00000
Resolution (Å)	50-1.80 (1.83-1.80)	50-2.60 (2.64-2.60)	50-2.75 (2.80-2.75)
Total/unique reflections	111,910/30,247	36,510/9,796	27,751/8,058
Completeness (%)	97.0 (95.7)	99.8 (99.6)	99.5 (99.8)
*R*_merge_ (%)	7.7 (43.4)	5.9 (41.3)	7.9 (43.8)
*I*/σ (*I*)	26.9 (4.1)	27.8 (4.0)	31.1 (4.0)
Refinement statistics			
Resolution (Å)	20.0-1.80	40.0-2.60	20.0-2.75
*R*_work_/*R*_free_ (%)	17.5/20.2	22.0/27.3	20.2/28.7
R.m.s. deviations from ideal			
Bond lengths (Å)	0.012	0.012	0.010
Bond angles (°)	1.49	1.52	1.50
Ramachandran plot (%)			
Favored	98.1	92.4	90.6
Allowed	1.9	7.6	9.4

To obtain an α2-Man_3_-bound complex, the *C*2 crystals of the ERGIC-53–CRD/MCFD2 binary complex were soaked in a solution containing 10 mM Ca^2+^ and 5 mM α2-Man_3_. The final model of the ternary complex refined to a resolution of 2.75 Å has an *R*_work_ of 20.2% and *R*_free_ of 28.7% (Table 1). We also determined a Man_2_-bound complex at 2.60 Å resolution with an *R*_work_ of 22.0% and *R*_free_ of 27.3% (Table 1). In the both structures, two loops comprised of residues 155–161 and 176–185 (termed Loops 1 and 2, respectively) of ERGIC-53, which were disordered in the Ca^2+^-free form (Figure 1B). These are unambiguously observed with Ca^2+^ ions coordinated thereto (Figure 1C). The sugar-binding site of ERGIC-53 is located in a pocket neighboring the Ca^2+^-binding site on the concave β-sheet as in VIP36 [30]. In particular, Asn156 directly interacts with the mannosyl ligand in the presence of the Ca^2+^ ions (Figure S1); thus, visualizing a Ca^2+^-dependent sugar-binding mode of ERGIC-53–CRD as in the case of VIP36 [30].

The absence of Ca^2+^ ions resulted in disorder of the sugar-binding loops of ERGIC-53–CRD in complex with MCFD2, as previously observed in the Ca^2+^-free form of ERGIC-53–CRD alone [27]. In contrast, the present crystal structure along with the previously reported crystal structures [28], [29] indicated that MCFD2 assumed an almost identical conformation in complex with ERGIC-53-CRD irrespective of the presence or absence of the Ca^2+^ ions in the sugar-binding site of ERGIC-53 (Figure S2). The Ca^2+^-dependent conformational disruption occurring specifically in the sugar-binding loops of the ERGIC-53–CRD/MCFD2 complex may explain how the complex releases its cargo on the secretory pathway.

### Sugar-binding site of ERGIC-53

**Figure 2:**
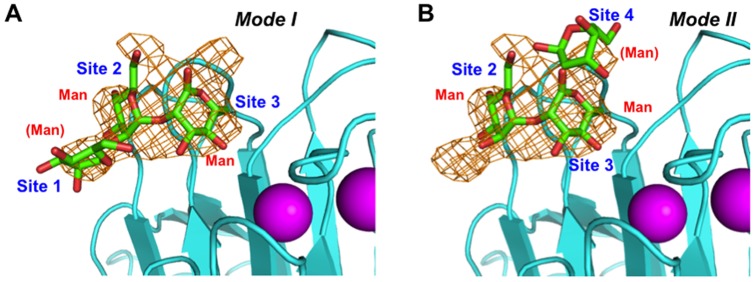
Sugar-binding site of ERGIC-53. Omit *F*_o_–*F*_c_ electron density map of α2-Man_2_ contoured at 2.0 σ. Additional electron densities flank the Manα1,2-Man moiety occupying the primary binding sites (sites 2 and 3), suggesting two alternative binding modes: (A) mode I and (B) mode II. The mannose residue occupying site 1 was modeled by superimposing the reducing-terminal mannose residue of the α2-Man_2_ ligand in the VIP36 crystal structure (PDB code: 2DUR) [30], which has a highest resolution (1.65 Å) among the L-type lectin-sugar complexes so far reported, while the modeling of the mannose residue accommodated in site 4 was based on the crystal structure of the non-reducing-terminal mannose residue of α2-Man_2_ bound in ERGIC-53 (PDB code: 4GKX) [31].

**Figure 3:**
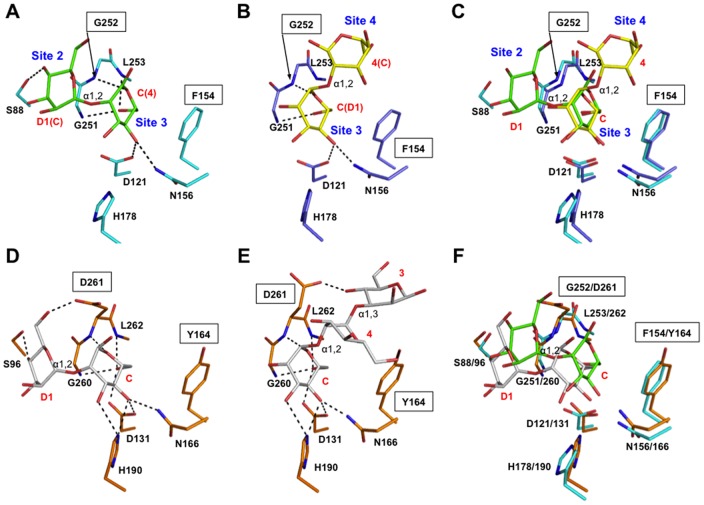
Comparison of sugar-binding sites of ERGIC-53 and VIP36. (A) Structure of the central α2-Man_2_-binding pocket (sites 2 and 3) of ERGIC-53-CRD (cyan, this study). (B) Structure of α2-Man_2_-binding site (3 and 4) of ERGIC-53-CRD (slate, PDB code: 4GKX) [31]. (C) Superposition between the two complexes of ERGIC-53–CRD. Assignments of the moieties of Man_9_GlcNAc_2_ are indicated in red characters. (D) VIP36–CRD (orange) complexed with α2-Man_2_ corresponding to Man(D1)-Man(C) (PDB code: 2DUR) [30]. (E) VIP36–CRD complexed with Man-α1,2-Man-α1,3-Man corresponding to Man(C)-Man(4)-Man(3) (PDB code: 2E6V) [30]. (F) Superposition between the Man(D1)-Man(C) bound complexes formed with ERGIC-53–CRD (cyan) and VIP36–CRD (orange). The variable residues among L-type lectins are indicated by boxes.

In the α2-Man_3_-bound ternary complex, we could identify electron densities corresponding to the ligand occupying four sites in the CRD (Figure 2; hereafter designated sites 1, 2, 3, and 4). Two mannose residues occupying sites 2 and 3 were clearly visible in the electron density map and could be traced as Man-α1,2-Man, indicating that these two sites compose the primary α2-Man_2_-binding pocket. The mannose residue that occupied site 3 interacted with Asp121 (Oδ-1 and Oδ-2), Asn156 (Nδ-2), Gly251 (N), Gly252 (N), and Leu253 (N) through extensive hydrogen bonds in the complex (Figure 3A), consistent with previous biochemical studies [11], [31], [32], [33]; mutations of Asp121 and Asn156 of ERGIC-53 abolished the binding capability to mannose and the cargo glycoproteins, FVIII and cathepsin Z-related protein. In addition, Phe154 exhibited a hydrophobic interaction with the C4, C5, and C6 atoms of this mannose residue. In site 2, the mannose residue forms a hydrogen bond between its 4-OH group and Ser88 (Oγ). In the α2-Man_2_-bound ternary complex, the two mannose residues were also accommodated in the sites 2 and 3 in the same fashion (Figure S3).

Intriguingly, the Man-α1,2-Man occupying the primary binding sites (sites 2 and 3) were flanked by ambiguous electron densities at sites 1 and 4 indicating the existence of an additional mannose residue in these low-affinity subsites (Figure 2A and 2B). The reducing terminal mannose residue occupying site 4 was also identified in the previously reported α2-Man_2_-bound binary complex structure (Figure 3B and 3C) [31]. These findings prompted us to propose two alternative modes of accommodation of α2-Man_3_ in the sugar binding pocket of the CRD of ERGIC-53 (Figure 2A and 2B). One mode employs sites 1, 2, and 3 (mode I), while the other involves sites 2, 3, and 4 (mode II): The latter corresponds to the previously reported ligand accommodation pocket of the VIP36 CRD, although there exists a significant difference between these two lectins in terms of detailed sugar-binding mode (*vide infra*). Such two-way modes in recognition of the same mannosyl ligand have been reported for concanavalin A [34].

### Structural basis for sugar-binding specificity of ERGIC-53

We previously showed that glucosylation or trimming of the D1 mannosyl arm affects its binding to VIP36–CRD [21]. To provide a structural basis for the broad sugar-biding specificity of ERGIC-53, we compared the detailed interaction modes between α2-Man_3_-bound human ERGIC-53 and canine VIP36 bound to mannosyl ligands [30]. In mode I, the 3-OH group of Man(D1) was situated outward with respect to the sugar binding pocket, enabling the Glcα1-3 linkage formation without steric hindrance (Figure 2A). However, this binding mode was not employed by the VIP36 CRD, because the reducing-terminal mannose residue, Man(4), extensively interacted with Tyr164 in site 4 rendering mode II exclusively predominant in comparison with the loose interaction in this site in ERGIC-53 (Figure 3E and S4) [30]. Even in mode II, the carbohydrate accommodation way in sites 2 and 3 is significantly different between ERGIC-53 and VIP36 (Figure 3F). First, the Man(C) residue in site 3 pocket is located closely adjacent to Loop 1 in ERGIC-53 to achieve hydrophobic interactions with Phe154. This phenylalanine corresponds to Tyr164 in VIP36, in which the side-chain Oη atom creates a hydrogen bond with the 6-OH group of the α1,2-linked Man(4) residue. Second, the α2-Man_2_ residues are less extensively immersed into the ERGIC-53 pocket in comparison with the sugar binding mode of VIP36. This is because Asp261 of VIP36 is substituted with Gly252 in ERGIC-53. The lack of the protruding aspartate side chain renders a sugar-binding pocket of ERGIC-53 significantly shallower (Figure S4). On the other hand, the Asp261 creates a hydrogen bond with the 6-OH group of the Man(D1) residue in VIP36. The two key residues Tyr164 and Asp261 in VIP36 are not conserved in ERGIC-53 (Phe154 and Gly252), explaining how VIP36 has higher affinity for high-mannose-type oligosaccharides than ERGIC-53 [22].

**Figure 4:**
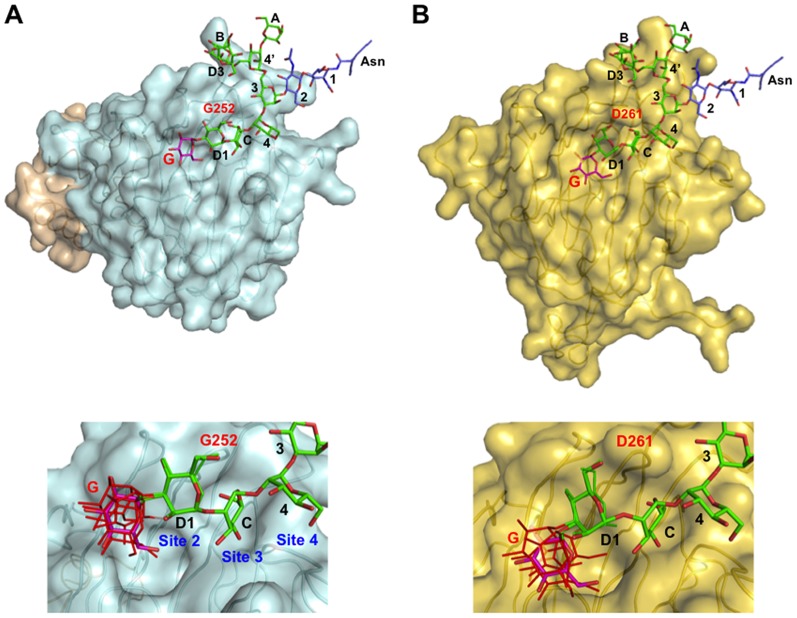
Structural models of L-type lectins with monoglucosylated high-mannose-type oligosaccharides. Surface models of (A) ERGIC-53–CRD/MCFD2 and (B) VIP36–CRD are shown with the glucose, mannose, and *N*-acetylglucosamine residues displayed in magenta, green, and slate stick models, respectively. To model Glc_1_Man_8_GlcNAc_2_, we selected Glc-α1,3–Man coordinates from an insect arylphorin glycoprotein [PDB code: 3GWJ (molecule D)] based on the torsion angle energy estimated using the PDB-CARE program [40]. Other typical Glc-α1,3–Man structures (PDB codes: 3GWJ (molecule A), 3O0W, 3OGV, and 3OG2) with energetically acceptable torsion angles were also superimposed on the ERGIC-53 (A, bottom) and VIP36 (B, bottom) complexes. These coordinates are indicated by thin stick models (glucose: red, mannose: green). The nomenclature of oligosaccharide residues of Glc_1_Man_8_GlcNAc_2_ are shown as in Figure 1A.

The crystallographic data suggested that a steric hindrance occurs between the terminal glucose residue and Glu98 of VIP36, when a glucose residue is modeled into the complex at the Man(D1) position through an α1-3-linkage (Figure 4B) [30]. Furthermore, our previous FAC data demonstrated that ERGIC-53–CRD does not discriminate between monoglucosylated and deglucosylated high-mannose-type glycans [22]. In mode II, Man(D1) residue on site 2 of ERGIC-53 is positioned toward the loop including Gly251, Gly252, and Leu253 due to lack of the bulky aspartate side chain. This structural arrangement enables ERGIC-53–CRD to accept glucosylation of the accommodated ligand's D1 arm without steric hindrance (Figure 4A). Consistent with the present crystallographic data, our previous FAC data demonstrated that a VIP36-CRD mutant in which Asp261 was replaced by glycine could bind monoglucosylated high-mannose-type glycans with comparable affinities for non-glucosylated ligands [22].

On the basis of these data we conclude that the sugar-binding specificities of L-type lectins are determined by the key glycine residue at the position 252 in human ERGIC-53. In addition, the two-way recognition modes of the trimannosyl glycotope by the ERGIC-53 CRD allows this lectin to accommodate the outermost glucose residue without severe specificity. The broad specificity of ERGIC-53 for high-mannose-type glycans may be beneficial for the efficient export of glycoproteins from the ER under ER stress conditions.

In summary, we determined the ternary complex structure comprising of ERGIC-53–CRD, MCFD2, and α2-Man_2_, or α2-Man_3_. Our results provide structural insights into the recognition mechanism of high-mannose-type glycoproteins by ERGIC-53 with broad specificity.

## Materials and Methods

### Crystallization, X-ray data collection, and structure determination

The purification of the binary complex of ERGIC-53−CRD (residues 31–269) and MCFD2 (residues 67–146) was performed as previously described [28]. The ERGIC-53/MCFD2 complex (7 mg/mL) was dissolved in 20 mM Tris-HCl (pH 7.5) and 2 mM CaCl_2,_ and the crystals were obtained in a buffer containing 20% PEG5000 monomethyl ether, 100 mM Bis-Tris (pH 6.5) on incubation at 16°C for 1 week. To obtain the sugar-bound ternary complexes, the crystal was soaked with this crystallization buffer containing 10 mM CaCl_2_ and 5 mM 2α-mannotriose or 10 mM 2α-mannobiose (Sigma-Aldrich) for 30 min and 5 h, respectively. α-D-mannopyranosyl-(1→2)-α-D-mannopyranosyl-(1→2)-α-D-mannopyranose (2α-mannotriose) was chemically synthesized [30] as shown in Figure S5. Subsequently, the crystals were cryoprotected with the soaking buffer supplemented with 20% glycerol. The crystals of the ternary complexes belonged to space group *C*2 with one complex per asymmetric unit and diffracted up to a resolution of 2.75 Å (α2-Man_3_-bound) and 2.60 Å (α2-Man_2_-bound). Diffraction data were processed using HKL2000 [35]. The crystal parameters are shown in Table 1.

The crystal structures of the ERGIC-53/MCFD2/α2-Man_2_ complexes were solved by the molecular replacement method using the program MOLREP [36] with the binary ERGIC-53–CRD/MCFD2 complex (Protein Data Bank code 3A4U) as a search model. On the basis of electron density maps, models were manually created using COOT [37]. The refinement procedure was performed using REFMAC5 [38]. The stereochemical quality of the final model was assessed using RAMPAGE [39]. The refinement statistics are summarized in Table 1. The molecular graphics were prepared using PyMOL (http://www.pymol.org/).

### Computer-aided model building

The model of the ERGIC-53/Man_8_GlcNAc_2_–Asn complex was created using coordinates of well-ordered high-mannose-type glycans on glycoprotein crystal structures as previously described [30]. Subsequently, the corresponding mannobiose residues on the ERGIC-53/MCFD2 complex were superimposed on each other. The model of monoglucosylated Glc_1_Man_8_GlcNAc_2_–Asn was created by the superimposition of the Glc-α1,3–Man disaccharide originating from a glycoprotein crystal structure [insect arylphorin (PDB code: 3GWJ, molecule D)], which has the most energetically preferred torsion angles among known three-dimensional structures (Figure 4 and Figure S6). The torsion angle energy was estimated using the PDB-CARE program [40] and the distribution map of Glc-α1,3–Man is shown in Figure S5.

## Supporting Information

Figure S1 **Close up view of the sugar and Ca^2+^-binding site of ERGIC-53.** Residues involved in sugar binding and Ca^2+^ coordination are shown in stick models. Ca^2+^-coordinating bonds are solid lines, whereas hydrogen bonds are dotted lines. Ca^2+^-binding loops are highlighted in orange as in Figure 1C.
(TIF)

Figure S2 **Structural comparison of ERGIC-53-CRD/MCFD2 crystal structures with different space group.** Superimposed ERGIC-53/MCFD2 structures are shown: ERGIC-53 [cyan (*C*2, PDB code: 3WHT) and green (*P*3_1_21, PDB code: 3A4U)] [28], MCFD2 [purple (*C*2) and yellow (*P*3_1_21)], Ca^2+^ ion [magenta (*C*2) and red (*P*3_1_21)].
(TIF)

Figure S3 **Electron density map of α2-Man_2_-bound ERGIC-53.** Omit *F*_o_–*F*_c_ electron density map of α2-Man_2_ contoured at 2.0 σ in the α2-Man_2_-bound complex.
(TIF)

Figure S4 **Surface models of sugar-binding sites of ERGIC-53 and VIP36.** Superposition of sugar-bound complexes showing their binding sites 2-4 in ERGIC-53 (A) and VIP36 (B).
(TIF)

Figure S5 **Synthesis of Man-α1,2-Man-α1,2-Man.** NIS: *N*-iodosuccinimide, TfOH: triflic acid, PhSH: thiophenol, OEt_2_: diethyl ether.
(TIF)

Figure S6 **Torsion angle distribution map of the Glc-α1,3–Man linkage.** The disaccharide torsion angles in the models were plotted in red (for the 3GWJ-derived model, molecule D) and black (for the others). Definitions of torsion angles are as follows: φ; O_5_-C_1_-O_1_-C′_x_, ψ; C_1_-O_1_-C′_X_-C′_X + 1_.
(TIF)
