# Supplementary figures and images for: Structural Basis for Disparate Sugar-Binding Specificities in the Homologous Cargo Receptors ERGIC-53 and VIP36
Source: PLoS One. 2014 Feb 3;9(2):e87963. doi: 10.1371/journal.pone.0087963 (PMC3912170; doi:10.1371/journal.pone.0087963)

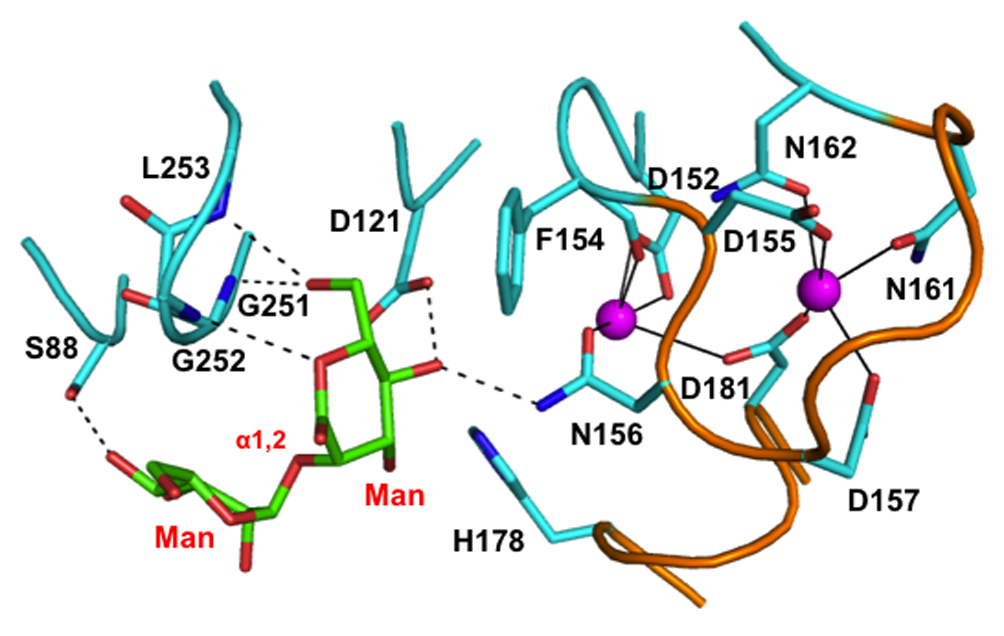

Supplement: Figure S1 — Close up view of the sugar and Ca2+-binding site of ERGIC-53. Residues involved in sugar binding and Ca2+ coordination are shown in stick models. Ca2+-coordinating bonds are solid lines, whereas hydrogen bonds are dotted lines. Ca2+-binding loops are highlighted in orange as in Figure 1C. (TIF) [file pone.0087963.s001.tif]

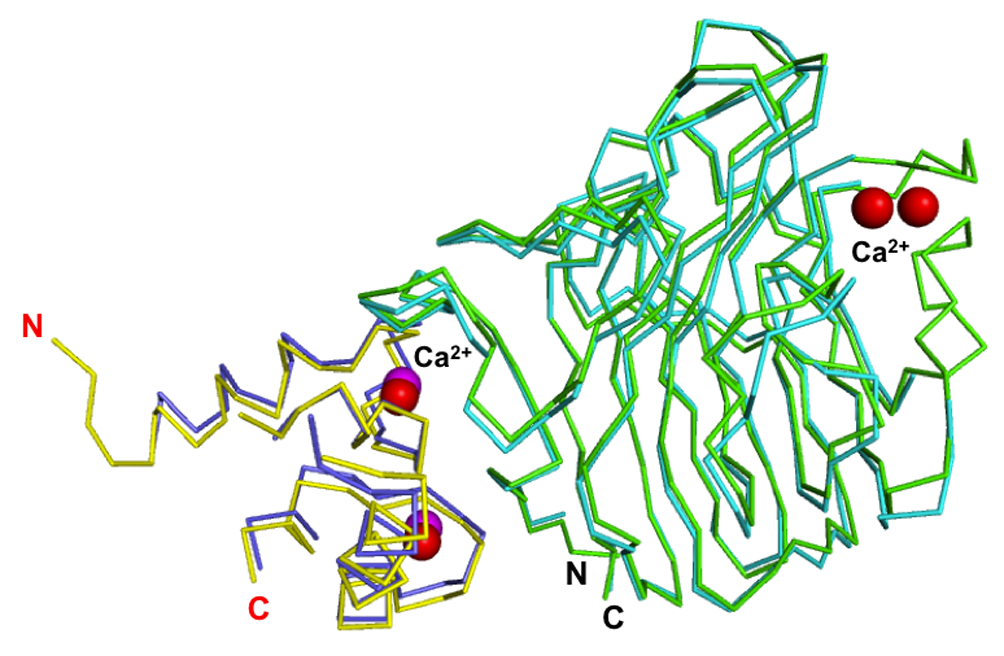

Supplement: Figure S2 — Structural comparison of ERGIC-53-CRD/MCFD2 crystal structures with different space group. Superimposed ERGIC-53/MCFD2 structures are shown: ERGIC-53 [cyan (C2, PDB code: 3WHT) and green (P3121, PDB code: 3A4U)] [28], MCFD2 [purple (C2) and yellow (P3121)], Ca2+ ion [magenta (C2) and red (P3121)]. (TIF) [file pone.0087963.s002.tif]

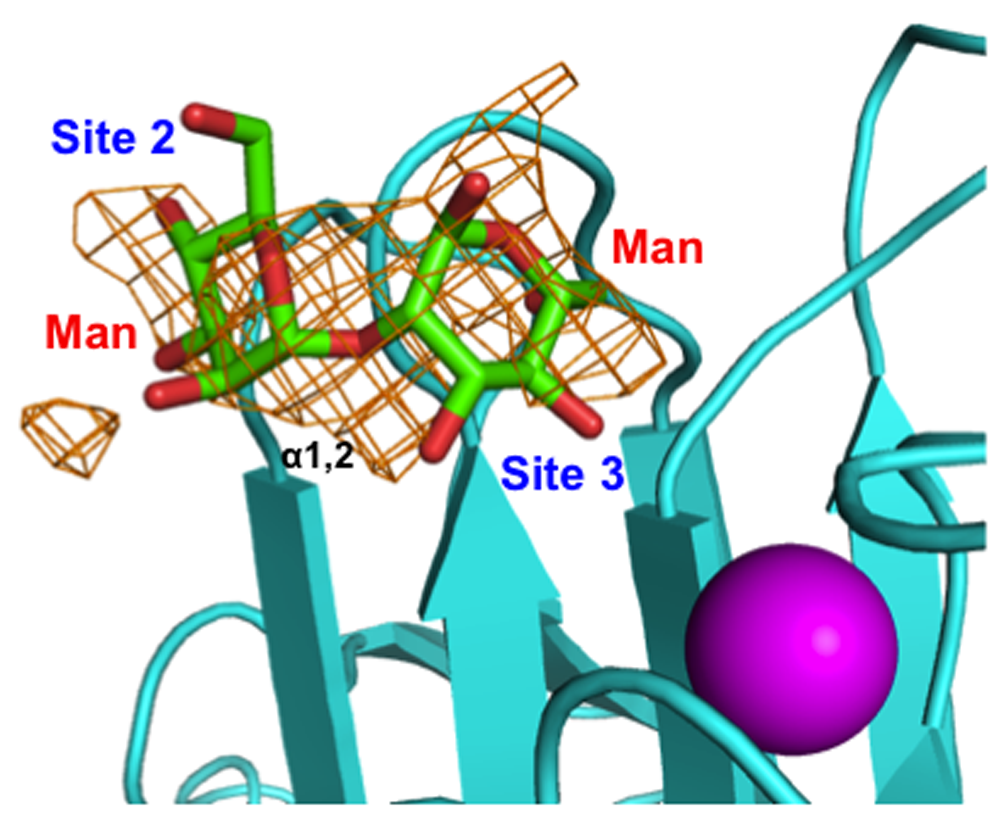

Supplement: Figure S3 — Electron density map of α2-Man2-bound ERGIC-53. Omit F o–F c electron density map of α2-Man2 contoured at 2.0 σ in the α2-Man2-bound complex. (TIF) [file pone.0087963.s003.tif]

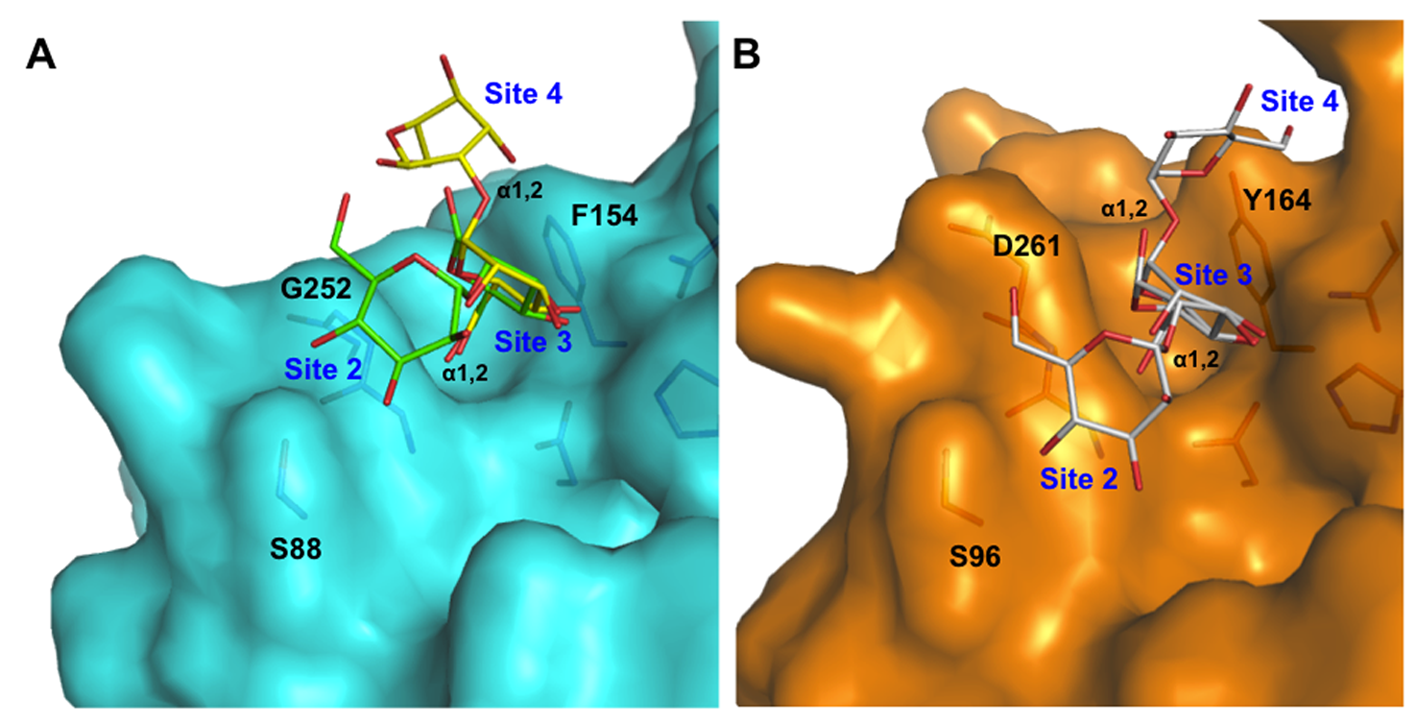

Supplement: Figure S4 — Surface models of sugar-binding sites of ERGIC-53 and VIP36. Superposition of sugar-bound complexes showing their binding sites 2-4 in ERGIC-53 (A) and VIP36 (B). (TIF) [file pone.0087963.s004.tif]

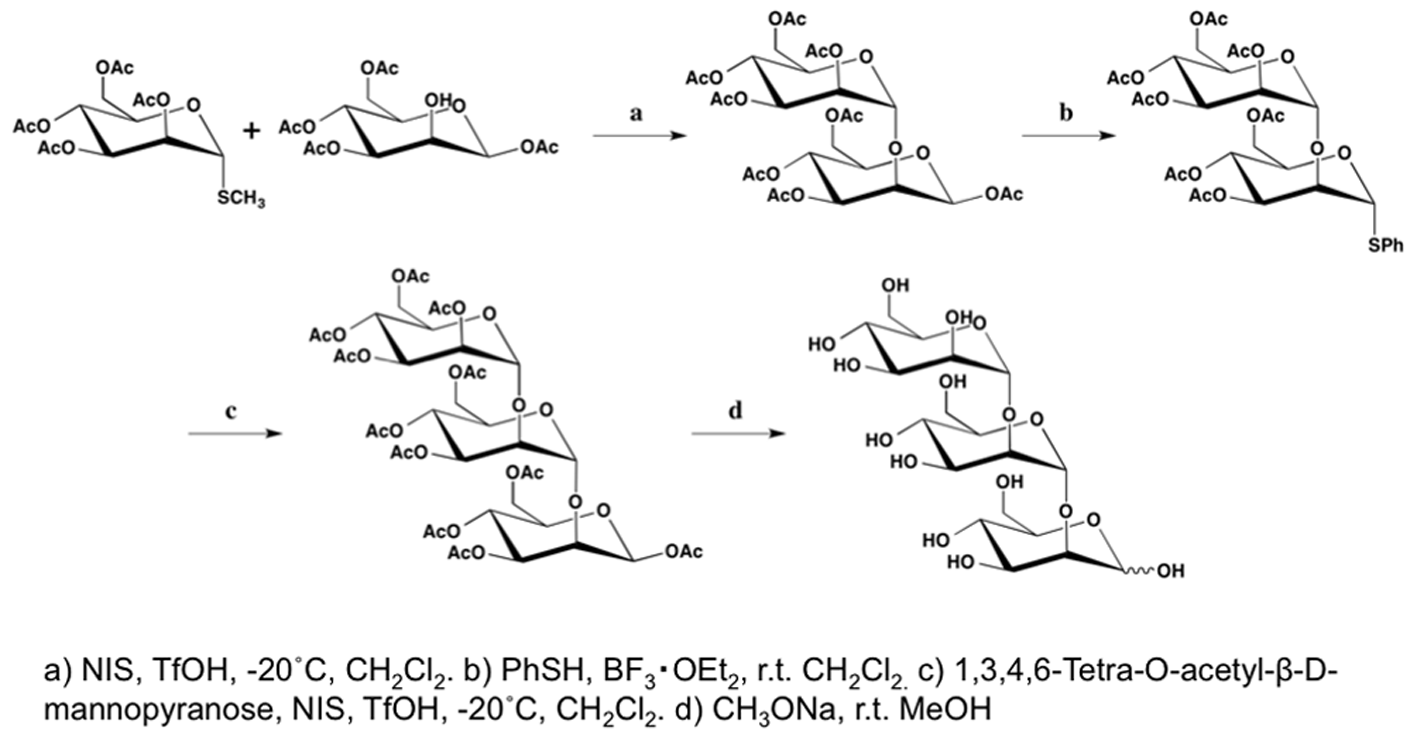

Supplement: Figure S5 — Synthesis of Man-α1,2-Man-α1,2-Man. NIS: N-iodosuccinimide, TfOH: triflic acid, PhSH: thiophenol, OEt2: diethyl ether. (TIF) [file pone.0087963.s005.tif]

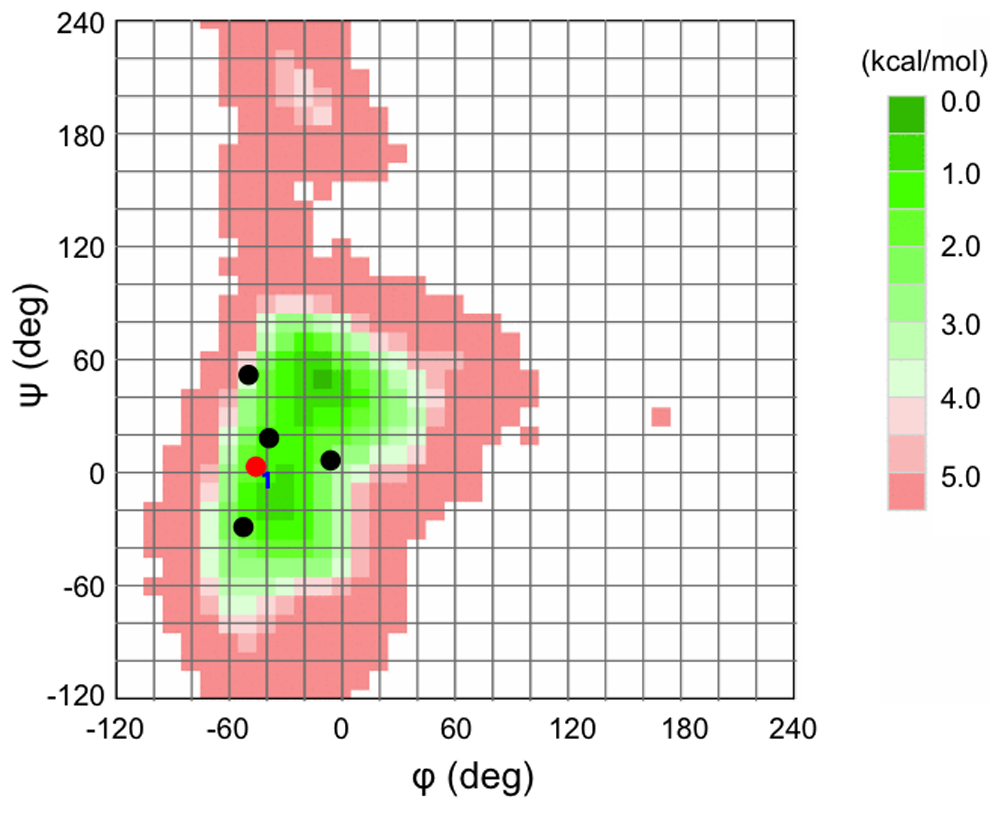

Supplement: Figure S6 — Torsion angle distribution map of the Glc-α1,3–Man linkage. The disaccharide torsion angles in the models were plotted in red (for the 3GWJ-derived model, molecule D) and black (for the others). Definitions of torsion angles are as follows: φ; O5-C1-O1-C′x, ψ; C1-O1-C′X-C′X + 1. (TIF) [file pone.0087963.s006.tif]
